# The value of LH maximum level in predicting optimal oocyte yield following GnRH agonist trigger

**DOI:** 10.3389/fendo.2023.1216584

**Published:** 2023-08-07

**Authors:** William Hao-Yu Lee, Kuan-Ting Lin, Yun-Chiao Hsieh, Tzu-Ching Kao, Ting-Chi Huang, Kuang-Han Chao, Mei-Jou Chen, Jehn-Hsiahn Yang, Shee-Uan Chen

**Affiliations:** ^1^ Department of Obstetrics and Gynecology, National Taiwan University Hospital, Taipei, Taiwan; ^2^ Infertility Center, Chien-Shin Hospital, Kaohsiung, Taiwan; ^3^ Taipei IVF Clinic, Taipei, Taiwan; ^4^ Livia Shangyu Wan Chair Professor of Obstetrics and Gynecology, College of Medicine, National Taiwan University, Taipei, Taiwan

**Keywords:** suboptimal trigger, LH max, GnRH-agonist trigger, OHSS, dual trigger

## Abstract

**Background:**

Risk factors associated with a suboptimal response to Gonadotropin-releasing hormone (GnRH) agonists include a high or low body mass index (BMI), prolonged use of oral contraceptive pills, and low luteinizing hormone (LH) levels on either the start or trigger days of controlled ovarian stimulation (COS). However, this approach may increase the need for a dual trigger and may also result in a higher incidence of ovarian hyperstimulation syndrome (OHSS) in hyper-responders. We aimed to investigate whether the maximum LH level during stimulation can serve as a predictive factor for achieving an optimal oocyte yield using the GnRH agonist trigger alone.

**Methods:**

We retrospectively reviewed all antagonist protocols or progestin-primed ovarian stimulation (PPOS) protocols triggered with GnRH agonist only between May 2012 and December 2022. Subjects were divided into three groups, depending on basal LH level and LH maximum level. The freeze-all strategy was implemented in all cycles: Group 1, consistently low LH levels throughout COS; Group 2, low basal LH level with high LH max level during COS; Group 3, consistently high LH levels throughout COS. The primary outcome was the oocyte yield rate. The secondary outcome includes the number of collected oocytes, suboptimal response to GnRH agonist trigger, oocyte maturity rate, fertilized rate, clinical pregnancy rate, ongoing pregnancy rate, and live birth rate. The pregnancy outcomes were calculated for the first FET cycle.

**Results:**

Following confounder adjustment, multivariable regression analysis showed that Group 1 (cycles with consistently low LH levels throughout COS) remains an independent predictor of suboptimal response (OR: 6.99; 95% CI 1.035–47.274). Group 1 (b = −12.72; 95% CI −20.9 to −4.55) and BMI (b = −0.25; 95% CI −0.5 to −0.004) were negatively associated with oocyte yield rate. Patients with low basal LH but high LH max levels had similar clinical outcomes compared to those with high LH max levels through COS.

**Conclusions:**

The maximum LH level during COS may serve as an indicator of LH reserve and could be a more reliable predictor of achieving an optimal oocyte yield when compared to relying solely on the basal LH level. In the case of hyper-responders where trigger agents (agonist-only or dual trigger) are being considered, we propose a novel strategy that incorporates the maximum LH level, rather than just the basal or trigger-day LH level, as a reference for assessing LH reserve. This approach aims to minimize the risk of obtaining suboptimal oocyte yield and improve overall treatment outcomes.

## Introduction

During controlled ovarian stimulation (COS) for *in vitro* fertilization (IVF) cycles, the most commonly used agent for inducing final oocyte maturation is human chorionic gonadotropin (hCG). However, for hyper-responders, using a high dose of hCG for triggering can increase the risk of ovarian hyperstimulation syndrome (OHSS). Gonadotropin-releasing hormone agonist (GnRH-a) can induce LH surge and trigger final oocyte maturation, providing an effective method for reducing OHSS risk in patients receiving a GnRH antagonist protocol or progestin-primed ovarian stimulation (PPOS) protocol (1, 2). Previous studies have shown that severe OHSS can be nearly completely prevented through GnRH-a trigger and freeze-all strategy (1, 3–5). There have been only a few case reports of OHSS in GnRH-a trigger and freeze-all cycles (6).

However, suppression of the hypothalamic–pituitary axis during or before COS may impact the response to the GnRH-a trigger. Lu et al. (7) found that a low LH level (<15 IU/L) at 12 h post-trigger was strongly associated with a decreased oocyte yield, which is known as a suboptimal trigger. In severe cases, empty follicle syndrome occurred in 0.5%–3.5% of GnRH-a trigger-only cycles (8, 9). Lu et al. (7) suggested that low basal LH level (<2.27 IU/L) was a useful predictor of a suboptimal response to GnRH-a trigger, and they proposed that a dual trigger with GnRH-a plus hCG could improve oocyte retrieval rates for GnRH-a suboptimal responders. Meyer et al. (10) reported that patients with very low endogenous serum LH levels on the trigger day had a 25% chance of a suboptimal LH surge, and they recommended GnRH-a trigger only for patients with LH > 0.5 IU/L on the trigger day and a dual trigger for patients with LH < 0.5 IU/L, which reduced the rate of suboptimal response from 5.2% to 0.2%. However, the addition of hCG in a dual trigger may increase the risk of OHSS (11).

Currently, basal LH levels or LH levels on the trigger day are useful predictors for suboptimal response to GnRH-a trigger and the decision of a dual trigger (7, 10, 12). However, LH levels usually fluctuate during the course of the follicular phase in women undergoing COS. The LH levels on day 2 of menstruation (basal LH level) or trigger day may not completely reflect the LH reserve of pituitary function. We defined the largest value of LH level during COS as LH maximum (LH max).

**Figure 1:**
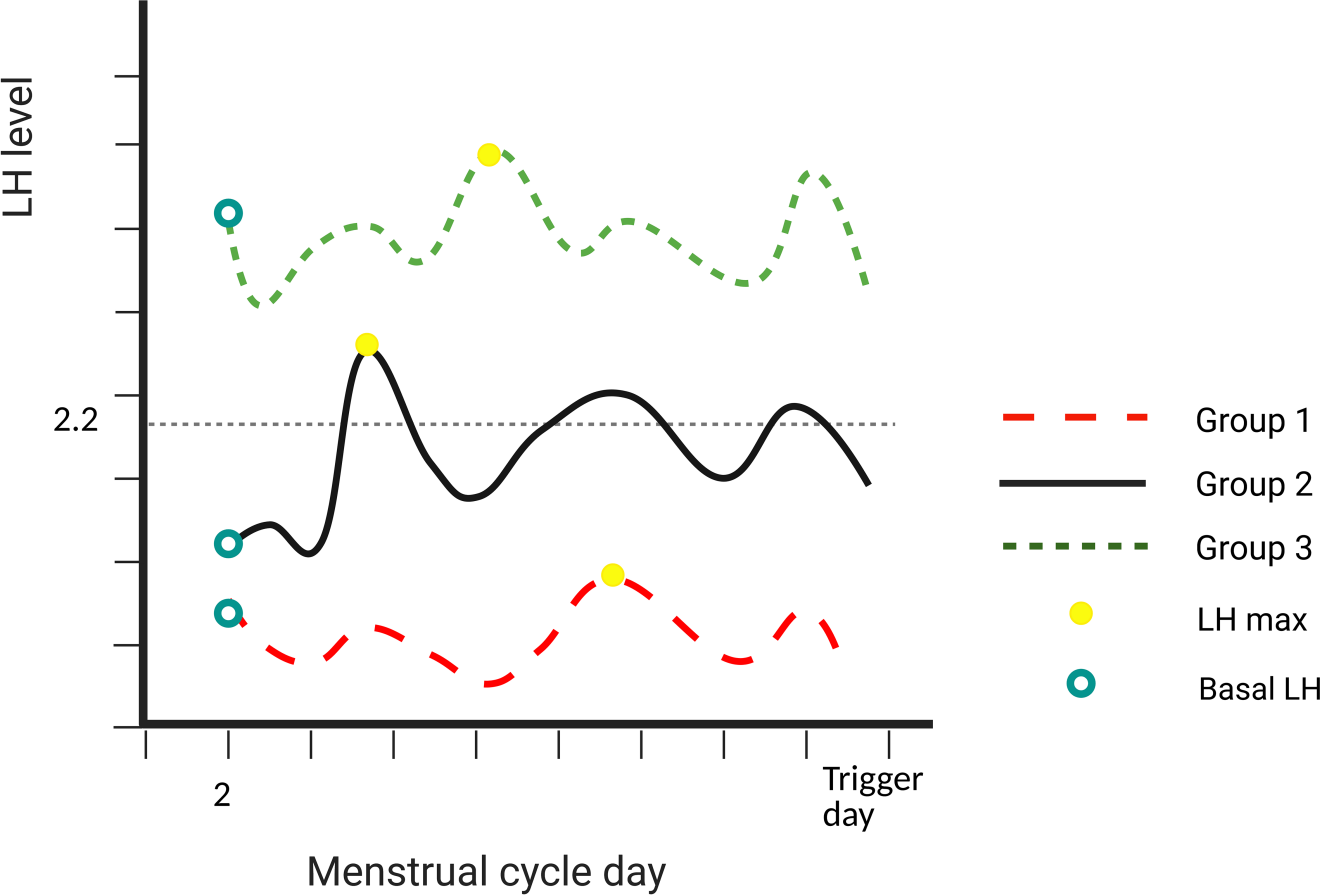
Description of the different groups. Group 1: basal LH level and LH max both ≤2.2 IU/L. Group 2: basal LH level ≤2.2 IU/L and LH max level >2.2 IU/L. Group 3: basal LH level and LH max both >2.2 IU/L. LH, luteinizing hormone.

We hypothesize that LH max may represent the LH reserve better than the basal LH level. In this study, we aimed to evaluate whether the value of LH max (**Figure 1**) could predict optimal oocyte yield in COS cycles triggered by GnRH-a only, especially for those women who hyper-respond to COS with lower basal LH levers but higher LH max levels.

## Materials and methods

### Study population and design

This is a retrospective, single-center, cohort study that includes IVF–intracytoplasmic sperm injection (ICSI) cycles (n = 1,077) in the National Taiwan University Hospital from May 2012 to December 2022. All cycles were only triggered with GnRH agonist, and all embryos were frozen to prevent OHSS. Patients with hypogonadotropic hypogonadism were excluded. Women with prior long-term oral contraceptive pill (OCP) usage before starting the COS cycle were also excluded.

### Ethical approval

This study was approved by the Institutional Review Board (IRB) of the National Taiwan University Hospital (Taipei City, Taiwan; serial number 202112224RINA). The IRB waived the requirement for informed consent owing to the retrospective nature of the study.

### Ovarian stimulation and GnRH agonist trigger

Ovarian stimulation was started on day 2 of the menstrual cycle with 100–300 IU/day of recombinant follicle-stimulating hormone (FSH) (rFSH: Gonal-F, Merck Pharmaceuticals, Darmstadt, Germany; Elonva, Merck Sharp & Dohme, Rahway, NJ, USA) or highly purified human menopausal gonadotropin (HMG) (HPHMG; Menopur, Ferring Pharmaceuticals, Saint-Prex, Switzerland). The dosage was adjusted according to the response of women undergoing COS by serial transvaginal ultrasound monitoring and blood estradiol level, which has been published previously (13, 14). Pituitary suppression was achieved with a daily 0.25 mg GnRH antagonist injection of ganirelix (Orgalutran, Merck Pharmaceuticals) starting on day 6 of ovarian stimulation or medroxyprogesterone acetate (MPA) 10 mg/day from the start of ovarian stimulation (3, 15). When at least three follicles of 17 mm or more were visible, triggering of final oocyte maturation was carried out with a single subcutaneous dose of GnRH agonist, 0.2 mg of triptorelin (Decapeptyl, Ferring Pharmaceuticals). Oocyte retrieval using the flushing method was performed at 35 to 36 h following the trigger. All follicles with a mean diameter of >12 mm were aspirated.

After oocyte pick-up (OPU), fertilization of the oocytes was performed by all IVF, 1/2 ICSI, or all ICSI, depending on semen parameters and physician preference. The freeze-all strategy was performed for all cycles.

### Embryo vitrification/thawing and transfer

In all agonist-triggered cycles, blastocysts were vitrified on day 5 or 6. The vitrification/thawing protocol was Cryotop method (Kitayazo, Japan) based on the method described by Kuwayama (16). Subsequently, thawed embryo transfer was scheduled based on the physician’s preference and the patient’s condition, using either a natural ovulatory cycle or a hormonal replacement cycle. For the hormone replacement cycle, endometrial preparation was initiated by stepwise increasing doses of oral estradiol valerate (Estrade 2 mg, Synmosa, Hsinchu, Taiwan) followed by the addition of 90 mg of vaginal progesterone (Crinone 8%; Merck Serono, Hertfordshire, UK) twice daily after at least 10–14 days of estrogen administration, as described previously (15). Blastocyst transfer was performed on the sixth day of progesterone administration. Clinical pregnancy was defined as the presence of fetal cardiac activity by transvaginal ultrasound at 7 weeks of gestation. Ongoing pregnancy was defined as progression beyond 12 weeks of gestation. Live birth rate was defined as the delivery of a live child after 24 weeks of gestation. The calculation of clinical pregnancy, ongoing pregnancy, and live birth rates were specifically focused on the first thawed embryo transfer cycle, where the highest-graded embryo was transferred.

### Stratification of patient groups

Several studies have indicated that a low basal LH level is a highly valuable marker for identifying suboptimal responders. Lu et al. (7) found that a basal LH level below 2.27 IU/L was the most significant marker for identifying suboptimal responders. Furthermore, Popovic-Todorovic et al. (17) reported that basal LH levels below 2 IU/L are associated with a 25.2% risk of suboptimal response.

To figure out whether LH max during COS plays a role in evaluating the optimal oocyte yield in the GnRH agonist trigger cycle, subjects were divided into three groups, depending on basal LH level (10th percentile in our cohort, 2.2 IU/L) and LH max level.

Group 1 included a cycle where basal LH level and LH max were both ≤2.2 IU/L. Group 2 included a cycle where basal LH level ≤2.2 IU/L and LH max level >2.2 IU/L. Group 3 included a cycle where basal LH level and LH max were both >2.2 IU/L. The schematic diagram is shown in **Figure 1**.

### Hormonal assessments

Serum estradiol, progesterone, FSH, and LH concentrations were measured for all patients using chemiluminescence assays (IMMULITE^®^ 2000; Siemens Healthineers, East Walpole, MA, USA) in every visit during ovarian stimulation, including day 2, day 6, day 8, and trigger day. Intra-assay and inter-assay coefficients of variation were 3.6% and 4.3%, respectively, for FSH; 4.8% and 10.7%, respectively, for LH; 6.7% and 9.7%, respectively, for E2; and 9.7% and 12.2%, respectively, for P4.

### Study outcomes

The primary outcome measure was oocyte yield rate, defined as the ratio of the total number of collected oocytes to the number of follicles (diameter > 12 mm) on the day of the trigger. The secondary outcome measures include the number of collected oocytes, suboptimal response to GnRH agonist trigger (defined as an oocyte yield below the <5th percentile (60.7% in our study)), oocyte maturity rate (the ratio of metaphase II oocytes to the number of denuded oocytes), fertilized rate (the ratio of normally fertilized oocytes (two distinct pronuclei (2PNs)) to the number of oocytes used for fertilization), clinical pregnancy rate (detection of a gestational sac during ultrasound), ongoing pregnancy rate (presence of fetal cardiac activity by transvaginal ultrasound at 9 weeks of gestation), and live birth rate (delivery of a live child after 24 weeks of gestation). The pregnancy outcomes were calculated for the first FET cycle in which the highest-graded embryo was transferred.

### Statistical analysis

SAS Version 9.4 (SAS Institute Inc.) was used for data analysis. For normally distributed data, the mean ± standard deviation was calculated for each variable. Non-normally distributed continuous data were reported as medians and interquartile ranges. Comparisons among groups were analyzed by analysis of variance (ANOVA) followed by an appropriate *post-hoc* test. Categorical variables were presented as frequencies, using percentages.

Multivariable logistic and linear regression analyses were conducted for adjustment of covariates to evaluate the suboptimal response, oocyte yield rate, mature oocyte rate, total collected oocyte, and clinical pregnancy. A p-value of less than 0.05 was statistically significant.

## Result

Between May 2012 and December 2022, a total of 1,077 antagonist or PPOS cycles were triggered only with GnRH agonists. Of these, 96 cycles were excluded from the analysis for the following reasons: 26 cycles used recombinant LH, which would have affected serum LH level measurement; 24 cycles involved a combination use of clomiphene; and 46 cycles had incomplete hormonal measurements. In total, 981 cycles (209 PPOS protocol and 772 antagonist protocol) were included in the final analysis.

### Baseline patient and cycle characteristics

Baseline characteristics, hormone profiles, and laboratory parameters are shown in **Tables 1**, **2**. The mean age, BMI, and anti-Mullerian hormone (AMH) were 34.8 ± 4.3 years, 22.1 ± 3.5 kg/m^2^, and 6.2 ± 4.1 ng/ml, respectively. The mean serum FSH and LH levels at the start of stimulation were 6.6 ± 2.8 and 5.3 ± 2.7 IU/L, respectively (**Table 2**).

**Table 1:** Baseline patient characteristics.

Parameter	N = 981
Age (mean ± SD)	34.8 ± 4.3
BMI (mean ± SD)	22.1 ± 3.5
AMH (mean ± SD)	6.2 ± 4.1
Indication	
Oocyte freezing	190 (19.4%)
Male factor	178 (18.1%)
Advanced maternal age	84 (8.6%)
Tubal factor	76 (7.8%)
Oocyte donor	50 (5.1%)
Preimplantation genetic diagnosis	48 (4.9%)
Uterine	12 (1.2%)
Idiopathic	72 (7.3%)

BMI, body mass index; AMH, anti-Mullerian hormone.

**Table 2:** Hormonal profiles and outcome.

Parameters	N = 981
Serum hormone levels at the start of ovarian stimulation	
FSH (mean ± SD)	6.6 ± 2.8
LH (mean ± SD)	5.3 ± 2.7
E2 (mean ± SD)	40.9 ± 24
Progesterone (mean ± SD)	0.4 ± 0.30
Outcome	
Follicles > 12 mm on the day of trigger	24.5 ± 10.2
Retrieved oocytes/case (mean ± SD)	23.7 ± 9.9
Oocyte yield rate (mean ± SD)	89.4% ± 13.7%
Maturity rate (mean ± SD)	83.8% ± 13.3%
Fertilization rate (mean ± SD)	64.7% ± 17.9%
Number of suboptimal response cycle	39 (3.9%)
Clinical pregnancy rate (95% CI)	46.8% (43.2–50.3)
Ongoing pregnancy rate (95% CI)	45.84% (42.3–49.4)
Live birth rate (95% CI)	41.04% (37.5–44.6)

FSH, follicle-stimulating hormone; LH, luteinizing hormone.

Regarding the indication of enrolled participants for COS in this study, oocyte freezing accounted for 190 cycles (19.4%) and male infertility for 178 cycles (18.1%), while preimplantation genetic screening (PGS) was performed in 48 cycles (4.9%). The remaining indications for COS are presented in **Table 1**.

On average, patients had 24.5 ± 10.2 follicles >12 mm in diameter on the day of trigger, and the mean number of retrieved oocytes was 23.7 ± 9.9. Oocyte maturity was calculated only for patients undergoing ICSI or 1/2 ICSI. The mean oocyte yield rate, maturity, and fertilization rate were 89.4% ± 13.7%, 83.8% ± 13.3%, and 64.7% ± 17.9%, respectively. Thirty-nine patients exhibited a suboptimal response to GnRH agonist trigger, defined as an oocyte yield rate below the <5th percentile (60.7% in our study). For the first frozen embryo following embryo freezing, clinical pregnancy, ongoing pregnancy, and live birth rates were 42.7%, 42.2%, and 37.9%, respectively (**Table 2**).

### Characteristics and outcomes after LH max stratification

Following stratification by basal LH and LH max, 17 women (1.8%) had an LH level ≤2.2 IU/L throughout the entire stimulation cycle before the trigger, 31 women (3.2%) had basal LH ≤2.2 IU/L with LH max >2.2 IU/L, and 933 women (95.1%) had an LH level >2.2 IU/L during the entire stimulation cycle before the trigger (**Table 3**).

**Table 3:** Demographic data and parameters of different groups.

Characteristics	Group 1	Group 2	Group 3	p-Value
Cycles, n	17	31	933	
Age at retrieval, years (mean ± SD)	30.9 ± 6.4	35.3 ± 3.6	34.8 ± 4.2	**<0.0001 ^a,b^**
Body mass index, kg/m^2^ (mean ± SD)	22.7 ± 3.0	26.1	21.9 ± 3.4	**<0.0001^b,c^**
AMH, ng/ml (mean ± SD)	6.1 ± 3.7	5.2 ± 3.7	6.2 ± 4.2	NS
COS protocols				NS
Antagonist	16	21	734	
PPOS	1	10	199	
Serum hormone level at start of ovarian stimulation (mean ± SD)				
FSH, IU/L (mean ± SD)	5.0 ± 2.93	6.3 ± 6.9	6.73 ± 2.6	**0.0369**^a^
LH, IU/L (mean ± SD)	1.4 ± 0.3	1.9 ± 0.3	5.5 ± 2.7	**<0.0001^a,b,c^**
E2, pg/ml (mean ± SD)	49.4 ± 59.4	45.1 ± 27.6	40.6 ± 22.8	NS
Progesterone, ng/ml (mean ± SD)	0.5 ± 0.28	0.5 ± 0.2	0.4 ± 0.3	NS
LH max, IU/L (mean ± SD)	1.6 ± 0.4	5.4 ± 3.5	7.0 ± 4.7	**<0.0001^a,b^**
Fertilizing method				NS
All IVF, n	2	1	34	
1/2 ICSI, n	5	10	161	
All ICSI, n	9	13	535	
Outcome				
Retrieved oocytes/cycle (mean ± SD)	24.0 ± 8.7	23.3 ± 10.9	23.7 ± 9.9	NS
Oocyte yield rate (mean ± SD)	78.2 ± 22.8	89.4 ± 15.1	89.6 ± 13.4	**0.0031^a,b^**
Suboptimal response cycle, n (%)	4/17 (23.5%)	2/31 (6.45%)	34/933 (3.64%)	**0.0002**
Maturity rate (mean ± SD)	84.4 ± 10.1	86.1 ± 14.5	83.7 ± 13.4	NS
Fertilize rate (mean ± SD)	71.4 ± 13.8	63.2 ± 23.7	64.7 ± 17.7	NS
Clinical pregnancy rate, n (%)	11/16 (68.7%)	14/24 (58.3%)	334/730 (45.7%)	NS
Ongoing pregnancy rate, n (%)	10/16 (62.5%)	14/24 (58.3%)	329/730 (45.1%)	NS
Live birth rate, n (%)	9/16 (56.3%)	11/24 (45.8%)	296/730 (40.55%)	NS
Complication				
OHSS rate, n (%)	0/17 (0%)	0/31 (0%)	3/933 (0.32%)	NS

AMH, anti-Mullerian hormone; COS, controlled ovarian stimulation; PPOS, progestin-primed ovarian stimulation; FSH, follicle-stimulating hormone; LH, luteinizing hormone; IVF, in vitro fertilization; ICSI, intracytoplasmic sperm injection; OHSS, ovarian hyperstimulation syndrome.

a Significant between Groups 1 and 3.

b Significant between Groups 1 and 2.

c Significant between Groups 2 and 3.

Bold means the p value <0.05, which is significant.

Women with LH levels ≤2.2 IU/L during the whole stimulation cycle (Group 1) had the youngest age at retrieval and the lowest basal FSH level (p < 0.05), but no significant differences were observed in AMH (6.1 ± 3.7 *vs.* 5.2 ± 3.7 *vs.* 6.2 ± 4.2), basal estradiol level (49.4 ± 59.4 *vs.* 45.1 ± 27.6 *vs.* 40.6 ± 22.8), or progesterone level (0.50 ± 0.28 *vs.* 0.51 ± 0.27 *vs.* 0.42 ± 0.31) among the three groups.

Group 1 had a significantly lower oocyte yield rate compared to the other two groups. However, oocyte yield rates did not differ significantly between Group 2 and Group 3. The suboptimal response rate was significantly higher in Group 1 (23.5%) compared to Group 2 (6.5%) and Group 3 (3.6%).

There were no significant differences in the COS protocols, maturity rate, fertilization rate, fertilizing method, clinical pregnancy rate, ongoing pregnancy rate, and live birth rate among the groups, and the OHSS rate did not differ significantly among the three groups.

We conducted a multiple regression analysis to further investigate the association between the three groups and the impact on oocyte yield rate, maturity rate, and fertilization rate. After adjustment for all potential explanatory variables, such as age at retrieval, BMI, basal FSH, and E2 level, Group 1 (b = −12.723; 95% CI, −20.9 to −4.55; p = 0.002) and BMI (b = −0.254; 95% CI, −0.5 to −0.004; p = 0.46) were still significantly negatively correlated with oocyte yield rate compared to Group 2. As shown in **Table 4**, none of the groups were associated with the number of retrieved oocytes. Only age at retrieval (b = −0.583; 95% CI, −0.72 to −0.44; p < 0.0001) and basal FSH level (b = −0.709; 95% CI, −0.92 to −0.49; p < 0.0001) remained significantly associated with the number of retrieved oocytes in the multiple regression model. The maturity rate and fertilization rate did not differ between the three groups after adjusting for all confounding variables.

**Table 4:** Factors predicting oocyte yield using multiple linear regression analysis for “oocyte yield rate”, “retrieved oocytes per cycle”, “maturity rate” and “fertilize rate”.

Independent variable	Oocyte yield rate			Retrieved oocytes/cycle			Maturity rate			Fertilize rate		
	Standard coefficient b	95% CI	p-Value	Standard coefficient b	95% CI	p-Value	Standard coefficient b	95% CI	p-Value	Standard coefficient b	95% CI	p-Value
Age at retrieval	−0.151	−0.35~0.05	0.143	**−0.583**	**−0.72~−0.44**	**<0.0001**	0.078	−0.12~0.28	0.446	0.026	−0.267~0.320	0.859
BMI	**−0.254**	**−0.50**~**−0.004**	**0.046**	−0.050	−0.22~0.12	0.566	0.092	−0.16~0.34	0.472	0.136	−0.235~0.506	0.473
Basal FSH level	0.031	−0.28~0.34	0.842	**−0.709**	**−0.92~−0.49**	**<0.0001**	0.130	−0.18~0.44	0.412	−0.311	−1.003~0.379	0.376
Basal E2 level	−0.031	−0.07~0.01	0.098	0.001	−0.02~0.03	0.913	−0.027	−0.06~0.01	0.151	0.003	−0.056~0.063	0.909
Group 1 *vs.* Group 2	**−12.723**	**−20.90~−4.55**	**0.002**	−3.458	−9.07~2.16	0.227	−0.555	−9.30~8.19	0.901	8.800	−2.769~20.370	0.136
Group 3 *vs.* Group 2	−1.073	−6.06~3.91	0.672	0.378	−3.05~3.80	0.828	−2.152	−7.42~3.12	0.423	2.534	−5.037~10.156	0.511

BMI, body mass index; FSH, follicle-stimulating hormone.

Bold means the p value <0.05, which is significant.

We utilized multiple logistic regression to compare the effects among three groups and investigate the impact of LH max and other critical variables on the suboptimal response of oocyte pick-up, clinical pregnancy, ongoing pregnancy, and live birth rates. After adjustment for all variables that may be related to suboptimal response, Group 1 still had a significantly higher risk of suboptimal response when compared to Group 2 (OR: 6.99; 95% CI, 1.035–47.274; p = 0.046). Groups 2 and 3 continued to have no difference in any clinical outcomes **Table 5**.

**Table 5:** Factors predicting oocyte yield using multiple logistic regression analysis for “suboptimal response”, “clinical pregnancy”, “ongoing pregnancy”, and “live birth”.

Independent variable	Suboptimal response			Clinical pregnancy			Ongoing pregnancy			Live birth		
	aOR	95% CI	p-Value	aOR	95% CI	p-Value	aOR	95% CI	p-Value	aOR	95% CI	p-Value
Age at retrieval	1.03	0.96–1.12	0.395	**0.934**	**0.902–0.967**	**<.0001**	**0.936**	**0.905–0.969**	**0.0002**	**0.932**	**0.899–0.965**	**<.0001**
BMI	1.05	0.97–1.15	0.225	0.977	0.936–1.020	0.2881	0.979	0.938–1.022	0.3293	0.996	0.953–1.041	0.8513
Basal FSH level	1.04	0.97–1.12	0.226	**0.835**	**0.764–0.913**	**<.0001**	**0.839**	**0.767–0.917**	**0.0001**	**0.841**	**0.767–0.** **922**	**0.0002**
Basal E2 level	0.99	0.98–1.01	0.735	0.995	0.988–1.002	0.1454	0.994	0.987–1.001	0.0793	0.993	0.981–1.005	0.2382
**Group1** *vs.* **Group 2**	**6.99**	**1.035–47.274**	**0.046**	1.628	0.370–7.167	0.5193	0.866	0.218–3.438	0.8379	1.186	0.287–4.895	0.8137
Group 3 *vs.* Group 2	0.67	0.144–3.135	0.6137	0.665	0.279–1.586	0.3574	0.650	0.273–1.549	0.3307	1.013	0.426–2.407	0.9763
Group 2 (reference)	1			1			1			1		

BMI, body mass index; FSH, follicle-stimulating hormone.

Bold means the p value <0.05, which is significant.

These results demonstrate that in patients undergoing GnRH antagonist or PPOS protocol with GnRH agonist trigger only, LH max level and BMI should be considered to achieve optimal oocyte yield outcomes, but they are not associated with clinical pregnancy, ongoing pregnancy, and live birth rates.

## Discussion

To the best of our knowledge, this is the first study to investigate the role of maximum LH level (LH max) during COS in GnRH agonist trigger cycles. Previous studies had indicated that low baseline LH level and low trigger day LH level are risk factors for suboptimal response to GnRH agonist triggering (7, 10, 18). However, there were 36.3% of cycles in our cohort, in which the maximum LH level was not at the start day or trigger day (**Supplementary Material**). It is not advisable to assess these patients based solely on LH levels obtained from a single day. The LH levels may fluctuate during COS. Those may also be influenced by estrogen-negative or estrogen-positive feedback as well as exogenous progestin or GnRH antagonist. Assessment of LH level during every visit may provide more information for endogenous LH reserve. After adjustment of confounders, our data showed comparable oocyte yield rate and maturity rate between patients with low basal LH level and high LH max level (Group 2) and those with normal basal LH (Group 3). Patients with low basal LH and low LH max (Group 1) have significantly lower oocyte yields than Group 2. The LH max level would be a valuable reference to determine the GnRH agonist trigger. There was no difference in the clinical pregnancy outcomes in the three groups. Although there was a suboptimal response to the GnRH agonist trigger, the retrieved oocytes in Group 1 were still found to be competent for achieving clinical pregnancy.

### Risk factor for suboptimal trigger

Suboptimal response to GnRH agonist trigger mainly caused by suboptimal trigger was a frustrating phenomenon during COS, leading to low oocyte yield or even empty follicle syndrome. Recently, one systemic review reveals that the incidence of empty follicle syndrome and suboptimal response (post-trigger LH <15 IU/L) in cycles triggered with GnRH agonists is approximately 0.5%–3.4% (17, 19, 20). In our study, a suboptimal response is 3.9% defined as an oocyte yield rate <60% (the lower 5th percentile of overall oocyte yield rate), which is comparable to that reported in other studies (18, 20). The risk factors for suboptimal response to GnRH agonist trigger had been reported as low or high BMI, low basal LH level or low trigger day LH level, prolonged stimulation, higher gonadotropins dose, long-term use of OCP, and hypogonadotropic hypogonadism (20). However, the determination of GnRH agonist or a dual trigger remains elusive.

### Basal LH level and trigger day LH level

Several studies attempted to explore the efficacy of GnRH agonist trigger, which focused on baseline LH or trigger day LH (7, 10, 17–19, 21). Chen et al. (18) reported that serum LH level <15 IU/L at 12 h post-trigger with GnRH agonist was associated with a dramatically lower oocyte yield (38.3%), compared to those with >15 IU/L (62.5%). Chang et al. (21) disclosed that patients with a low baseline LH <1 IU/L had a 13.3% risk of failure to GnRH agonist trigger, while that risk was reduced to 1.8% if the LH level was ≥2 IU/L. A lower LH level on the trigger day was also associated with an increased incidence of post-trigger serum LH <15 IU/L (10, 19). A low post-trigger LH level or low oocyte yield from the GnRH agonist trigger could be compensated by additional exogenous hCG. Meyer et al. (10) suggested a dual trigger for patients with low LH (<0.5 IU/L) on the day of the trigger. However, frequent administrations of hCG in the GnRH triggering cycles may increase the risk of OHSS.

Recent studies indicated that basal LH level was a better predictor for the efficacy of the GnRH agonist trigger than the trigger day LH (7, 17). Lu et al. (7) found that basal LH <0.5, 1.0, 1.5, 2.0, 2.5, 3.0, and 4.0 IU/L was associated with a 45.4%, 29.3%, 18.3%, 10.2%, 6.7%, 4.98%, and 3.5% of the risk for suboptimal response, respectively. They indicated that a cutoff basal LH level (<2.27 IU/L) was the single most valuable predictor (with area under the curve of 0.805) of a suboptimal response to the GnRH agonist trigger. They proposed a dual trigger for those patients with a cutoff basal LH level <2.27 IU/L. In our study, we defined the cutoff value by the 10th lower percentile of all basal LH levels, which was 2.2 IU/L, which was similar to the cutoff value by Lu et al. (7). We suggest maximum LH level during COS, representative of endogenous LH reserve, as a reference to determine agonist trigger or a dual trigger. Whether this strategy will achieve optimal oocyte yield and reduce OHSS deserves more prospective studies.

### Oocyte retrieval with flushing

In our center, oocyte pick-up is routinely performed by junior fellows using follicle flushing and curetting. The oocyte yield rate is also a criterion of junior fellow evaluations. The value of follicular flush in oocyte retrieval remains controversial (22, 23). Some investigators demonstrated that follicular flush increased oocyte yield (22, 24). However, some investigators found that follicular flushing failed to increase the oocyte yield rate (23). In addition, follicular curettage during oocyte retrieval may increase oocyte yield without sacrificing oocyte quality (25, 26). The average oocyte yield rate (89.4%) in the present study was higher than that of previous other reports (68.26%) (7). These may partly explain our relatively high oocyte yield rate in GnRH agonist-trigger patients. The value of flushing and curettage in the GnRH agonist trigger cycles may deserve further investigation.

### Limitations

The number of cases in both Group 1 and Group 2 was relatively low in our cohort. In our center, it is not our routine practice to administer OCPs for withdrawal of bleeding prior to COS. Additionally, long-term OCP users were excluded from our study. The use of oral contraceptive pills as pretreatment prior to COS appeared to be more common in a previous article by Meyer et al. (2019). It is worth noting that the use of OCP may result in lower basal LH levels.

In our retrospective study, in order to account for confounding factors, we performed a multiple regression analysis to examine the relationship between the three groups (taking into account the impact of LH max) and their effects on oocyte yield rate, maturity rate, and fertilization rate.

The primary weakness of our study is its retrospective design, which introduced heterogeneity and confounders due to the varied implementation of stimulation protocols by different providers. To better evaluate the LH max, a randomized prospective trial comparing Group 1, Group 2, and Group 3 should be conducted in the future.

The post-trigger LH level was not routinely measured in our center. Our primary outcome was essential for oocyte yield rate, but no sufficient data on post-trigger LH levels were available. Conversely, serum LH profile was obtained in every revisit during ovarian stimulation and could be more comprehensive to evaluate endogenous LH reserve. Furthermore, most subjects did not undergo preimplantation genetic testing (PGT), which would control for aneuploidy, and the embryo transfer number and quality were not reported in this study.

Future research should investigate the relationship between post-trigger LH levels and LH max levels. Additional data are necessary to determine a definitive serum LH max cutoff level for optimal GnRH agonist triggering, which may vary based on a patient’s baseline LH level and BMI. It would also be valuable to analyze a cohort of patients with a euploid embryo confirmed by PGS.

## Conclusions

Using GnRH agonists for triggering final oocyte maturation is effective and safe for hyper-responders in antagonist protocol or PPOS. However, a small fraction of patients with a suboptimal response or risk of OHSS should be carefully assessed. We provide a new strategy with a reference of LH max (not only basal LH) to assess the LH reserve. We suggest measurements of LH levels during COS and consider LH max to evaluate the endogenous LH reserve. A suboptimal response to GnRH agonist trigger may be anticipated in a low LH max (consistently low LH levels throughout COS) and high BMI patient, and an individualized approach is warranted for a dual trigger. However, although there was low oocyte yield in Group 1, the clinical pregnancy outcomes were not compromised.

In hyper-responders with a low baseline LH level but high LH max level, which indicates an adequate LH reserve, triggering with a GnRH agonist alone may yield similar rates of oocyte yield and maturation while reducing the risk of OHSS. Therefore, this hypothesis may warrant further investigation to potentially reduce the need for a dual trigger and decrease the risk of OHSS.

## Data availability statement

The raw data supporting the conclusions of this article will be made available by the authors, without undue reservation.

## Ethics statement

This study was approved by the IRB of the National Taiwan University Hospital (Taipei City, Taiwan; serial number 202112224RINA). Written informed consent for participation was not required for this study in accordance with the national legislation and the institutional requirements.

## Author contributions

WH-YL wrote the main manuscript text, performed the statistical analysis, and prepared the figure. K-TL prepared the tables. Y-CH, T-CK, T-CH, K-HC, M-JC, and J-HY collected all patients’ data. S-UC was the head of the project, and he revised the final manuscript and ensured that the tables and figure were correct according to the collected data.
