# Supplementary material for: The value of LH maximum level in predicting optimal oocyte yield following GnRH agonist trigger
Source: Front Endocrinol (Lausanne). 2023 Aug 7;14:1216584. doi: 10.3389/fendo.2023.1216584 (PMC10441777; doi:10.3389/fendo.2023.1216584)
Supplement: Supplementary file 1 [file Table_1.docx]

Supplement material

Table 6.

Timing of LH max in different protocols

|  | Antagonist protocol (772) | PPOS protocol (209) | Total |
| --- | --- | --- | --- |
| LH max occurs at day 2 | 482 (62.4%) | 103 (49.2%) | 585(59.6%) |
| LH max occurs at trigger | 25 (3.2%) | 12 (5.7%) | 37(3.8%) |
| LH max occurs during COS | 265 (34.3%) | 94 (44.9%) | 359(36.5) |
